# Multiplexed targeted mRNA profiling of alcohol-related liver disease reveals stage-specific dysregulation of signaling pathways

**DOI:** 10.1007/s11033-026-11955-z

**Published:** 2026-06-09

**Authors:** Dusan Rasic, Maja Thiele, Peter Andersen, Aleksander Krag, Sönke Detlefsen

**Affiliations:** 1 Department of Pathology, Odense University Hospital, Odense, Denmark; 2 Department of Clinical Research, Faculty of Health Sciences, University of Southern Denmark, Odense, Denmark; 3 Fibrosis, fatty liver and steatohepatitis research center Odense (FLASH), Department of Gastroenterology and Hepatology, Odense University Hospital, Odense, Denmark

**Keywords:** Alcoholic liver disease, Gene expression profiling, Alcoholic cirrhosis, LGALS3, S100A4

## Abstract

**Introduction:**

Alcohol-related liver disease (ALD) remains a leading indication for liver transplantation and a major cause of alcohol-attributable deaths worldwide. The molecular events driving extracellular matrix (ECM) accumulation are incompletely understood. In this study, we aimed to investigate stage-specific gene expression signatures in ALD.

**Methods and results:**

Liver biopsies from 50 adults with prior alcohol overuse were selected: No fibrosis (controls (F0), n = 10), mild/moderate fibrosis (F1–F2, n = 19), and advanced fibrosis/cirrhosis (F3–F4, n = 21). We performed multiplexed targeted profiling of 760 mRNAs, followed by analysis of differentially expressed genes (DEGs) and gene set enrichment analysis. Selected targets were validated on the protein level using immunohistochemistry. Mild/moderate fibrosis versus controls showed 80 DEGs (p < 0.01), 63 of which were downregulated. MLXIPL and FURIN, both involved in glucose and lipid metabolism, were downregulated. Pathways involved in ECM formation were enriched. Advanced fibrosis vs. controls showed 187 DEGs (p < 0.01), of which 94 were upregulated. These included fibrogenic and inflammatory mediators such as TGFB1, COL1A1/2, TIMP1, LGALS3, and S100A4. Genes involved in fatty acid, steroid, and lipid metabolism were significantly repressed. Advanced versus mild/moderate fibrosis showed 211 DEGs (p < 0.01), with an increase in TGF-β-driven ECM remodeling and inhibition of lipid and fatty-acid metabolism pathways. LGALS3 and S100A4 mRNA levels correlated with protein expression (R ≥ 0.41, p < 0.01).

**Conclusion:**

ALD is characterized by early downregulation of metabolic genes and upregulation of ECM formation, followed by coordinated induction of the TGF-β-centered fibrogenic pathway and further suppression of metabolic pathways. We identified stage-specific molecular alterations associated with ALD progression and LGALS3 and S100A4 as candidate biomarkers.

**Supplementary Information:**

The online version contains supplementary material available at 10.1007/s11033-026-11955-z.

## Introduction

Alcohol consumption is a major public health concern worldwide, with an estimated 2.3 billion people currently consuming alcohol [1]. A substantial share of alcohol-attributable mortality is due to chronic liver damage, with recent data indicating that approximately 4.8% of the world’s population suffers from alcohol-related liver disease (ALD), while ALD accounted for about 21% of all alcohol-attributable deaths (≈ 607,000 deaths globally) [1, 2]. In many high-income countries, ALD is the dominant indication for liver transplantation, especially as viral hepatitis-related cirrhosis declines [3].

ALD is characterized by a spectrum of hepatic changes and injuries, from steatosis to alcohol-related steatohepatitis, fibrosis, and ultimately cirrhosis [4]. In the fibrotic stage, chronic injury triggers activation of hepatic stellate cells (HSCs) and their transformation into myofibroblasts, leading to excessive deposition of collagen and extracellular matrix (ECM) components [5–7]. This results in the development of scar tissue that starts in the centrilobular region and progressively disrupts liver architecture, with fibrotic strands forming around central veins or between central veins and portal tracts – known as bridging fibrosis – and advancing from mild fibrosis to advanced fibrosis [8]. Cirrhosis marks the end-stage of ALD, where fibrous septa encircle nodules of regenerating hepatocytes or remnants of lobules, leading to irreversible architectural disruption. Clinically, cirrhosis is associated with portal hypertension and a higher risk of liver-related events and decompensation (ascites, variceal bleeding, encephalopathy), significantly elevating morbidity and mortality [5]. However, only a subset of heavy drinkers progress to advanced fibrosis and cirrhosis [9], but those who do face dramatically worse outcomes [10].

Despite extensive research into liver fibrogenesis, important gaps in our molecular and transcriptomic understanding of fibrosis progression in human ALD remain. Recent advances have also enabled transcriptomic profiling from formalin-fixed, paraffin-embedded (FFPE) tissues, including liver [11]. In metabolic dysfunction–associated steatotic liver disease (MASLD), large-scale studies have profiled hepatic gene expression across the full histological spectrum [12, 13], revealing insights into stage-specific pathways. For ALD, comparable transcriptomic data across disease stages is still sparse. Most studies to date have examined static snapshots of disease, often focusing on either early ALD versus end-stage cirrhosis, or on extreme phenotypes such as severe alcoholic hepatitis, rather than capturing the continuum of fibrosis development [14–16]. As a result, the stepwise changes in gene expression and signaling pathways that drive the transition from initial perivenular fibrosis to bridging fibrosis and cirrhosis are not fully delineated. Our understanding of when and how specific fibrogenic pathways (e.g., TGF-β signaling, inflammatory cascades, matrix remodeling genes) become dominant during progression is incomplete.

We hypothesized that ALD progression would be marked by distinct transcriptional profiles reflecting stage-specific activation of fibrogenic and inflammatory pathways. To enhance our understanding of the molecular changes occurring during ALD progression, we analyzed gene expression in remaining FFPE tissue from liver biopsies archived after initial use for standard pathology diagnosis, spanning the full spectrum of histological stages, from normal liver to manifest cirrhosis. In the present study, we aimed to identify stage-specific dysregulated mRNAs and pathways across the histological spectrum of human ALD.

## Materials and methods

We conducted a retrospective, single-center study examining gene expression to improve our understanding of the molecular changes occurring during ALD progression. We analyzed gene expression in FFPE liver biopsies from a cohort of 50 individuals spanning the full histological spectrum, from normal liver to advanced fibrosis.

The study included patients with prior alcohol overuse, defined as more than 24 g and 36 g per day for women and men, respectively, for more than 1 year. Additional inclusion criteria were age 18–75 years, informed consent to undergo a liver biopsy, no obvious signs of decompensated liver cirrhosis, no competing liver disease etiology, and abstinence from alcohol at the time of biopsy. The patients were investigated for ALD at Odense University Hospital (OUH), Odense, Denmark. A further ten liver biopsies without fibrosis (stage F0) were included as controls. All patients were identified based on fibrosis stage and had been included in previous studies [17, 18].

### Biopsy samples

Liver biopsies were performed percutaneously by experienced operators using suction needle biopsy (17G Mengini needle; Hepafix; Germany) after ultrasound examination. All biopsies were formalin-fixed and paraffin-embedded for pathologic evaluation. The biopsies were histochemically stained (hematoxylin-eosin (HE), Sirius red, reticulin, periodic acid-Schiff (PAS) with diastase, iron, copper) and immunohistochemically stained (CD38, CK7, ubiquitin) as part of the routine diagnostic panel and deemed adequate if they contained at least six portal tracts and had a length of at least 10 mm, unless regenerative nodules were present.

An experienced hepatopathologist (SD) evaluated all biopsies according to the NASH Clinical Research Network (NASH-CRN) Activity Score [8]. He assigned steatosis grades as S0 (< 5% hepatocytes with macrovesicular steatosis), S1 (5–33%), S2 (> 33–66%), and S3 (> 66% hepatocytes with macrovesicular steatosis) [8]. Lobular inflammation was scored as I0 (no inflammatory foci), I1 (< 2 inflammatory foci per 200x field), I2 (2–4 inflammatory foci per 200x field), and I3 (> 4 inflammatory foci per 200x field). Hepatocellular ballooning was scored B0 (none), B1 (few ballooned hepatocytes), and B2 (many ballooned hepatocytes or portal/prominent ballooning). Fibrosis stage was scored as F0 (no fibrosis), F1 (perisinusoidal or portal/periportal fibrosis), F2 (perisinusoidal and portal/periportal fibrosis), F3 (bridging fibrosis), and F4 (cirrhosis).

### RNA extraction

FFPE sections of 10 μm thickness were cut from the blocks and collected in 1.5 mL centrifuge tubes for RNA isolation. Number of sections depended on the size of the liver biopsy measured in mm. At least 100 mm^2^ total tissue area was included from all biopsies. The sections were prepared and processed according to the Prosigna^®^ Breast Cancer Prognostic Gene Signature Assay instructions (2016-09 LBL-C0223-06). Total RNA was extracted using High Pure FFPE RNA Isolation kit (Roche Diagnostics GmbH, Mannheim, Germany, 06650775001), according to the Prosigna^®^ protocol with one modification – d-limonene was used instead of xylene. Limonene was added to the samples, followed by centrifugation at 16,100 G for 2 min to dissolve the paraffin. Then, limonene was removed. After RNA extraction, RNA concentration was measured by Thermo Scientific™ NanoDrop™ One^C^ Spectrophotometer (Thermo Scientific) to assess RNA concentration (≥ 12.5 ng/µL) and purity (A260/A280 ratio between 1.7 and 2.3). A master mix was made using the Reporter CodeSet and hybridization buffer. The master mix was gently mixed by inversion and briefly centrifuged. A 12-tube strip was labelled, and the following reagents were added to each strip: 8 µL master mix, 5 µL of the extracted RNA sample, and 2 µL Capture ProbeSet. If the RNA concentration exceeded 20 ng/µL, they were diluted with molecular grade RNase- and DNase-free water (Sigma-Aldrich, 7732-18-5). If the RNA concentration was insufficient to obtain 100 ng total RNA, 5 µL of undiluted extracted RNA was used. Afterwards, hybridization assay was performed according to NanoString protocol. The RNA samples were stored at −80 °C. Conventional integrity metrics such as RIN or DV200 were not determined since the nCounter platform does not require high-integrity RNA, as it relies on direct hybridization of short probe pairs to target sequences, without cDNA synthesis or amplification, which makes it inherently robust to the RNA fragmentation characteristic of FFPE-derived material [19].

Quality control (QC) was performed according to manufacturer guidelines and included assessment of internal control probes, background signal, and normalization factors (e.g., housekeeping genes) using *NanoTube* R package (version 1.8.0) [20]. Predefined QC thresholds were applied to ensure data reliability. Two samples failed imaging QC due to a field-of-view (FOV) count below the manufacturer-defined threshold of 75%, Supplementary Table S1), unrelated to RNA quality or hybridization performance, and were therefore excluded for all downstream analyses. All remaining samples passed all predefined QC thresholds.

### Differential gene expression analysis

Expression levels of 760 mRNAs across 51 annotated pathways involved in development of fibrosis were assessed using the nCounter^®^ Human Fibrosis v2 Panel (NanoString Technologies, Seattle, WA). Chemical positive and negative controls and ten internal reference (housekeeping) genes for data normalization were included in this panel. Hybridized probes were bound to the optical cartridge, and the cartridge was scanned on the nCounter Digital Analyzer (NanoString Technologies). The raw data with digital counts were exported to the nSolver v4.0 software (NanoString) for downstream quality analysis according to the instructions provided in the nSolver manual. *NanoTube* R package [20] was used for raw data normalization using the geometric mean of internal chemical negative controls, positive controls, and 10 housekeeping genes (ACAD9, ARMH3, CNOT10, GUSB, MTMR14, NOL7, NUBP1, PGK1, PPIA, RPLP0). The statistical *limma* R package [21] was used for differential expression analysis across fibrosis stages. Linear models were developed and adjusted for covariates, namely, age, sex, BMI, and diabetes mellitus status. To correct for multiple comparisons, a P-value cutoff was used. As target genes were not randomly selected, standard techniques to correct for multiple comparisons were not used, as suggested by NanoString Technologies. We chose a stringent P-value cutoff of 0.01 to reduce the chance of false-positive results.

### Gene set enrichment analysis (GSEA)

We examined significant differentially expressed genes (DEGs) to determine if they were enriched in specific gene sets or pathways available in the *Reactome* database. GSEA was performed using the *gsePathway* function from the *ReactomePA* package in R [22–24]. The ranked gene list was based on t-statistic across fibrosis stages. Enrichment was assessed against the *Reactome* database for human (*hsa*), using Benjamini-Hochberg (BH) correction for multiple testing, with a false discovery rate (FDR) < 0.10, a minimal gene set size of 10, and a maximal gene set size of 500.

### Immunohistochemistry (IHC) and digital image analysis (DIA)

To investigate the protein-level expression of key targets identified through *limma*-based analysis of DEGs, we performed IHC on sections cut from the FFPE liver biopsies. Specifically, IHC was performed to detect galectin 3 (encoded by LGALS3*)* and S100A4 proteins, each of which had been implicated in critical biological pathways relevant to disease progression. Three to four-micron sections were cut and mounted on FLEX IHC Microscope slides and stained with HE. Sections were dried at room temperature and baked at 60 ˚C for 60 min before IHC. Protocols for IHC staining, including antibodies, dilutions, incubation times, and epitope retrieval procedures, are specified in Table 1.

**Table 1:** Antibodies used for IHC analysis

Antigen	Platform	Epitope retrieval	Dilution	Company and product ID	Type
Galectin-3	Discovery	HIER-CC1, 32 min, 100˚ C	1:100	Cell Marque, AC-0361 A	Rabbit monoclonal
S100A4	Discovery	HIER-CC1, 32 min, 100˚ C	1:1000	Abcam, ab27957	Rabbit polyclonal

CC1, cell conditioning solution 1. HIER, heat-induced epitope retrieval

The stained slides were scanned using a Hamamatsu NanoZoomer 2.0eHT whole slide scanner (Hamamatsu Photonics, Hamamatsu, Japan) at 20x magnification. Tissue regions were automatically detected using *OpenSlide* [25]. This enabled precise segmentation of tissue areas, ensuring that subsequent quantification of the IHC expression of selected proteins was performed exclusively within biologically relevant regions. Tissue region masks were quality checked and imported into QuPath digital image analysis software version 0.5.0 [26], and a pixel classifier was developed for each of the corresponding stains. Detailed description of the parameters used is specified in Supplementary Table S2. For each IHC marker, the ratio of positive area vs. total biopsy area was calculated.

### Statistical analysis

Summary statistics were generated for demographic and clinical variables. For continuous variables, comparisons were made between groups, and when ANOVA assumptions were met, one-way ANOVA was performed; otherwise, the Kruskal-Wallis test was performed. For categorical variables, comparisons were likewise made between all groups. Since sample sizes per group were low, the assumption of expected values ≥ 5 for the Chi-squared test was not met for any of the comparisonsion of expected, and thus, Fisher’s exact test was used. To assess the relationship between protein and gene expression, correlation analyses were performed using Spearman correlation, comparing DIA of IHC with mRNA counts. P-values < 0.05 were considered statistically significant. All analyses were performed using R version 4.3.3.

## Results

### Cohort characteristics

The study cohort included 50 individuals across fibrosis stages F0 to F4, which we grouped according to disease severity: 10 had no liver fibrosis and served as a control group, 19 participants had mild/moderate fibrosis (Kleiner fibrosis stages 1 or 2), and the remaining 21 cases had advanced fibrosis (Kleiner fibrosis stages 3 or 4). Median age was higher in the fibrosis groups (*p* < 0.01), and while the proportion of males increased with fibrosis severity (50% in controls, 68% in mild/moderate, 86% in advanced), the difference was not statistically significant. Low-density lipoprotein (LDL) cholesterol decreased with fibrosis progression (*p* = 0.037), while aspartate aminotransferase (AST) and gamma-glutamyl transferase (GGT) levels were significantly elevated in advanced fibrosis (*p* = 0.009 and *p* < 0.001, respectively). Histological evaluation showed increased lobular inflammation with higher fibrosis severity (*p* < 0.001). Storage duration differed significantly across groups (*p* = 0.006), with controls having longer storage times than both the mild/moderate and advanced fibrosis groups, which were comparable. This difference is mainly explained by a change in our clinical approach introduced in 2016 for patients with steatotic liver disease. In a previous study, we found by elastography that no patients with liver stiffness < 6.0 kPa had advanced fibrosis [18]. Based on this, liver biopsies were no longer routinely performed in patients with liver stiffness below 6.0 kPa, resulting in a marked reduction in biopsies from patients with fibrosis stage F0. Other clinical characteristics, including BMI, diabetes prevalence, and other liver enzyme levels, did not differ significantly between groups (Table 2).

**Table 2:** Patient and liver biopsy characteristics

*N* = 50	No fibrosis (*n* = 10)	Mild/moderate fibrosis (*n* = 19)	Advanced fibrosis (*n* = 21)	*P*-value
Age (years)	45.9 (10.6)	60.7 (8.0)	57.2 (7.4)	< 0.001
Sex				
Male	5 (50.0%)	13 (68.4%)	18 (85.7%)	0.109
Female	5 (50.0%)	6 (31.6%)	3 (14.3%)	
BMI	25.9 (3.8)	30.3 (8.0)	30.5 (8.5)	0.253
Diabetes, n (%)				
Yes	10 (100.0%)	12 (63.2%)	14 (66.7%)	0.085
No	0 (0.0%)	7 (36.8%)	7 (33.3%)	
Smoking, n (%)				
Never smoked	2 (20.0%)	5 (26.4%)	3 (14.3%)	0.107
Smoker	7 (70.0%)	7 (36.8%)	6 (28.6%)	
Previously smoked	1 (10.0%)	7 (36.8%)	12 (57.1%)	
Biochemistry				
LDL (mmol/L)	3.5 (1.0)	2.7 (1.1)	2.5 (0.9)	0.037
HDL (mmol/L)	1.4 (0.4)	1.2 (0.3)	1.2 (0.4)	0.252
Total cholesterol (mmol/L)	5.5 (1.2)	4.9 (1.1)	4.5 (1.1)	0.072
ALK (U/L)	72.5 (24.0)	90.8 (32.5)	93.0 (33.0)	0.165
AST (U/L)	25.3 (14.6)	28.3 (17.1)	31.2 (8.7)	0.009
ALT (U/L)	18.9 (6.9)	31.4 (23.4)	28.3 (17.7)	0.165
Bilirubin (µmol/L)	7.0 (3.8)	8.0 (3.8)	11.2 (8.4)	0.406
GGT (U/L)	21.7 (9.6)	50.5 (62.7)	86.0 (63.7)	< 0.001
Histological scores				
Steatosis grade, n (%)				
0	10 (100.0%)	12 (63.2%)	12 (57.1%)	0.067
1	0 (0.0%)	4 (21.0%)	8 (38.1%)	
2	0 (0.0%)	3 (15.8%)	1 (4.8%)	
3	0 (0.0%)	0 (0.0%)	0 (0.0%)	
Ballooning grade, n (%)				
0	10 (100.0%)	16 (84.2%)	15 (71.4%)	0.195
1	0 (0.0%)	3 (15.8%)	6 (28.6%)	
2	0 (0.0%)	0 (0.0%)	0 (0.0%)	
Lobular inflammation grade, n (%)				
0	9 (90.0%)	9 (47.4%)	2 (9.5%)	< 0.001
1	1 (10.0%)	9 (47.4%)	16 (76.2%)	
2	0 (0.0%)	1 (5.2%)	3 (14.3%)	
3	0 (0.0%)	0 (0.0%)	0 (0.0%)	
Sample age (median years and IQR)	9 (0.7)	5 (4.0)	4 (5.0)	0.006

BMI, body mass index. LDL, low-density lipoprotein. HDL, high-density lipoprotein. ALK, alkaline phosphatase. AST, aspartate aminotransferase. ALT, alanine aminotransferase. GGT, gamma-glutamyl transferase. Continuous variables are presented as mean ± standard deviation (SD).

### Differential gene expression across fibrosis stages

**Fig. 1:**
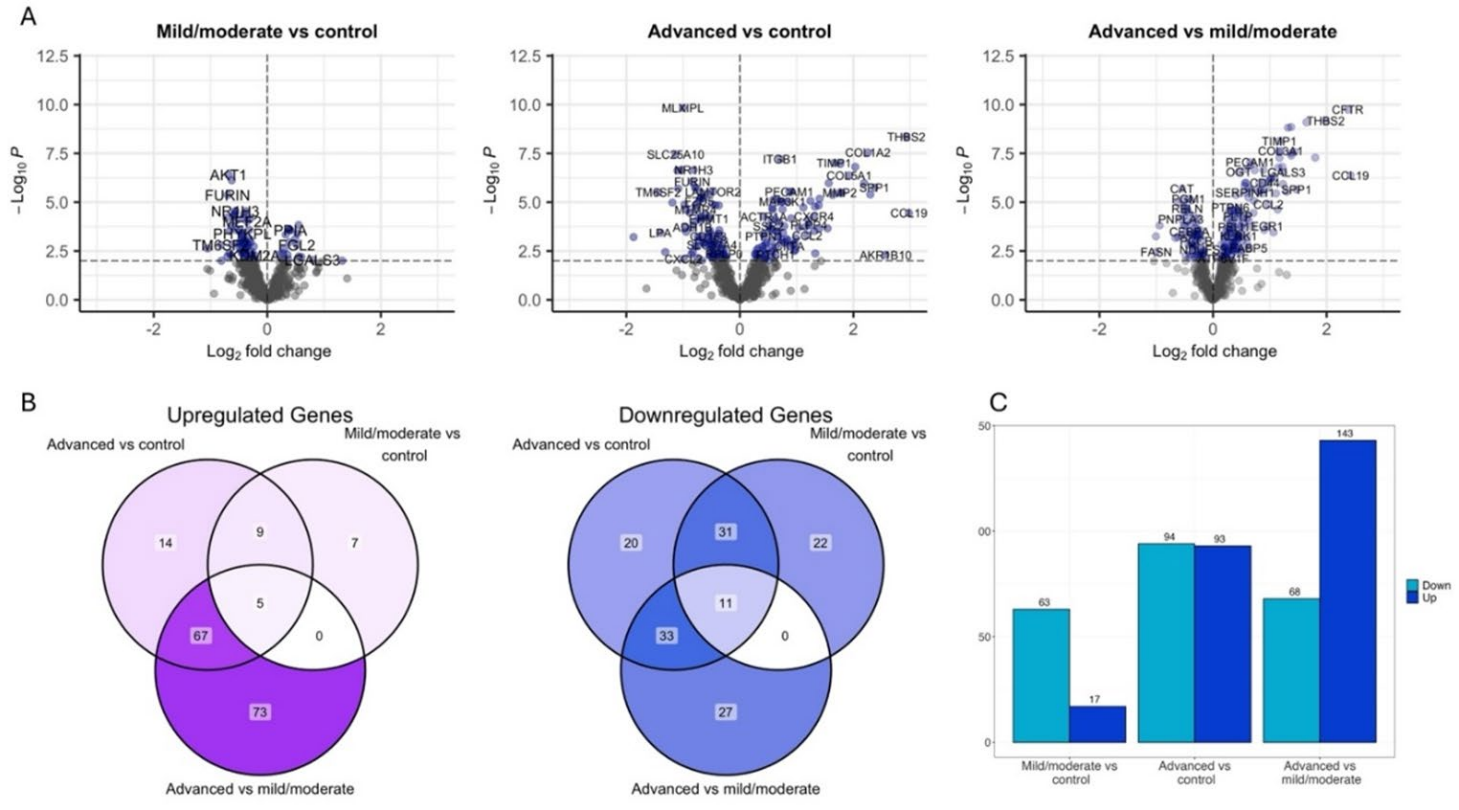
Differential gene expression in liver biopsies across fibrosis stages. (a) Volcano plots showing different comparisons – mild/moderate fibrosis vs. control, advanced fibrosis vs. control, and advanced vs. mild/moderate fibrosis. (b) Venn diagrams with upregulated and downregulated DEGs. (c) Bar chart with number of DEGs by different comparisons

The number of DEGs varied between disease severity groups. When comparing mild/moderate fibrosis with controls, we observed 80 DEGs (*p* < 0.01), 63 of which were downregulated (Fig. 1, Supplementary Table S3a-c). Notably, the AKT1 gene, a key regulator of cell survival and metabolic signaling, and MLXIPL and FURIN, both involved in glucose and lipid metabolism, were significantly downregulated (Table 3).

**Table 3:** Top differentially expressed genes (DEGs) in *Mild/moderate fibrosis vs. control* comparison

All upregulated DEGs				Top 30 downregulated DEGs			
Order	Gene	log_2_FC	P-value	Order	Gene	log_2_FC	P-value
1	CSNK1A1	0.551	0.0001	1	AKT1	−0.680	4.05E-07
2	ITGB1	0.390	0.0003	2	MLXIPL	−0.626	7.91E-07
3	COX7B	0.531	0.0004	3	FURIN	−0.697	4.27E-06
4	ACTR1A	0.324	0.0005	4	NR1H3	−0.560	2.83E-05
5	UBE2D2	0.489	0.001	5	SMAD4	−0.375	3.15E-05
6	SSR2	0.387	0.001	6	CYP27A1	−0.579	3.89E-05
7	FGL2	0.522	0.002	7	SORBS1	−0.574	4.09E-05
8	PRKAG1	0.297	0.003	8	RPS6KB2	−0.409	4.29E-05
9	BANF1	0.275	0.004	9	PRDX6	−0.585	5.22E-05
10	UBE2N	0.355	0.005	10	RORA	−0.719	6.33E-05
11	CTNNB1	0.218	0.005	11	ERP29	−0.421	6.83E-05
12	HLA-G	0.554	0.006	12	COX7C	−0.303	7.99E-05
13	WWC1	0.601	0.006	13	FZD5	−0.556	9.13E-05
14	HIKESHI	0.370	0.009	14	MEF2A	−0.355	0.0001
15	LGALS3	0.799	0.009	15	SLC25A10	−0.630	0.0002
16	CYP7A1	1.320	0.010	16	CCS	−0.542	0.0003
17	IFNGR1	0.282	0.010	17	IRF3	−0.479	0.0003
				18	LAMTOR2	−0.316	0.0003
				19	IL1RAP	−0.661	0.0003
				20	PHYKPL	−0.437	0.0004
				21	AP2S1	−0.453	0.0005
				22	EHMT1	−0.422	0.0005
				23	TRRAP	−0.311	0.0008
				24	SOD1	−0.393	0.001
				25	MAF	−0.414	0.001
				26	LRP6	−0.477	0.001
				27	XBP1	−0.445	0.001
				28	ATF7IP	−0.294	0.001
				29	TJP2	−0.431	0.001
				30	GRB10	−0.367	0.001

The data is ranked after the p-value. The complete list of significantly downregulated genes is shown in Supplementary Table 3. log_2_FC, log_2_-fold change.

In biopsies with advanced fibrosis, transcriptional alterations were more pronounced, with a greater number of DEGs compared to controls (Fig. 1, Supplementary Table S4a-c). We identified 187 DEGs, of which 94 were upregulated and 93 were downregulated. Key upregulated genes included TGF-β1-signaling pathway (e.g. TGFB1, CCN2, FURIN, THBS2, S100A4), ECM remodeling (e.g. COL1A1, COL1A2, TIMP1), and inflammation (LGALS3), while downregulation was observed for metabolic regulators (e.g. MLXIPL, AKT1, INSR) (Table 4).

**Table 4:** Top differentially expressed genes (DEGs) in *Advanced fibrosis vs. control* comparison

Top 30 upregulated DEGs				Top 30 downregulated DEGs			
Order	Gene	log_2_FC	P-value	Order	Gene	log_2_FC	P-value
1	THBS2	2.933	4.10E-09	1	MLXIPL	−1.004	1.56E-10
2	CFTR	3.235	8.25E-09	2	SLC25A10	−1.131	3.47E-08
3	COL1A2	2.257	2.72E-08	3	HSD11B1	−1.029	2.25E-07
4	ITGB1	0.709	6.68E-08	4	NR1H3	−0.812	2.28E-07
5	TIMP1	1.659	9.19E-08	5	RORA	−1.101	2.34E-07
6	COL3A1	1.770	1.06E-07	6	FURIN	−0.838	9.04E-07
7	LGALS3	2.033	1.48E-07	7	CYP27A1	−0.768	1.85E-06
8	COL5A1	1.927	4.07E-07	8	PRDX6	−0.792	1.90E-06
9	COL6A3	1.566	1.02E-06	9	APOC3	−1.086	1.96E-06
10	COL14A1	2.189	1.02E-06	10	TM6SF2	−1.436	3.02E-06
11	SPP1	2.370	1.72E-06	11	LAMTOR2	−0.473	3.24E-06
12	PECAM1	0.871	2.91E-06	12	AKT1	−0.667	3.50E-06
13	FGL2	0.916	2.94E-06	13	ERP29	−0.558	4.00E-06
14	MMP2	1.760	3.09E-06	14	PGM1	−0.696	6.02E-06
15	PDGFRB	1.799	3.27E-06	15	AP2S1	−0.680	6.11E-06
16	COL1A1	2.297	3.84E-06	16	PIK3R4	−0.636	6.54E-06
17	VIM	1.633	4.11E-06	17	FZD5	−0.715	9.15E-06
18	THBS1	1.403	6.44E-06	18	FNIP2	−1.189	1.02E-05
19	BAX	0.660	7.46E-06	19	PRKAB2	−0.773	1.31E-05
20	IFNGR1	0.586	7.93E-06	20	ADH4	−0.976	1.31E-05
21	S100A4	1.242	8.28E-06	21	RPS6KB2	−0.486	1.36E-05
22	MAP3K1	0.750	9.36E-06	22	SMAD4	−0.432	1.60E-05
23	CTBP2	0.847	9.51E-06	23	COX7C	−0.371	1.70E-05
24	CSNK1A1	0.732	9.68E-06	24	ACSM3	−0.939	2.45E-05
25	COL4A2	1.388	1.42E-05	25	MTMR4	−0.780	2.46E-05
26	COL4A1	1.324	1.65E-05	26	HMGCS2	−0.887	3.43E-05
27	WWC1	1.117	1.82E-05	27	TJP2	−0.622	4.50E-05
28	OGT	0.570	2.09E-05	28	ITPR2	−0.534	5.55E-05
29	GNB4	0.751	2.36E-05	29	CCS	−0.667	6.31E-05
30	CASP8	0.577	2.49E-05	30	ACACB	−0.720	6.35E-05

The data is ranked after the p-value. The complete list of significantly downregulated genes is shown in Supplementary Table 4. log_2_FC, log_2_-fold change.

We then investigated differences in key DEGs between biopsies with advanced fibrosis compared to mild/moderate fibrosis to assess which genes continue changing as fibrosis progresses (Fig. 1, Supplementary Table S5a-c). We found a total of 211 DEGs, of which 115 were also observed in the comparison between the advanced fibrosis and the control group. In total, 143 genes were upregulated, involved in TGF-β1 signaling pathway (TGFB1, CCN2, THBS2, S100A4, PDGFRB), ECM remodeling (COL1A2, COL4A1, COL4A2, TIMP1), and inflammation (e.g., LGALS3, CCL2) (Table 5). A total of 68 genes were downregulated, many of which were involved in metabolic regulation (e.g., MLXIPL) (Table 5).

Additionally, IL-8 (CXCL8), a key inflammatory mediator implicated in alcohol-associated hepatitis [27], was included in the targeted panel but did not pass predefined background filtering criteria in the majority of samples (78%), consistent with its generally low hepatic expression levels. Exploratory analysis of unfiltered expression data suggested a trend toward increased IL-8 expression with advancing fibrosis stage, particularly in samples with advanced fibrosis (Supplementary Fig. 1). As these data were not background-filtered, statistical comparisons were not performed, and this finding should be considered hypothesis-generating.

**Table 5:** Top differentially expressed genes (DEGs) in *Advanced vs. mild/moderate fibrosis* comparison

Top 30 upregulated DEGs				Top 30 downregulated DEGs			
Order	Gene	log_2_FC	P-value	Order	Gene	log_2_FC	P-value
1	CFTR	2.372	1.67E-10	1	CAT	−0.546	2.00E-06
2	THBS2	1.983	6.36E-10	2	PGM1	−0.432	6.95E-06
3	COL1A2	1.646	7.48E-10	3	HSD11B1	−0.532	7.74E-06
4	COL4A2	1.375	1.33E-09	4	MLXIPL	−0.378	8.51E-06
5	COL4A1	1.319	1.44E-09	5	APOC3	−0.618	1.01E-05
6	TIMP1	1.167	6.97E-09	6	ABCB11	−0.497	1.68E-05
7	COL3A1	1.192	2.21E-08	7	RELN	−0.454	2.17E-05
8	PDGFRB	1.440	2.51E-08	8	SLC25A10	−0.501	2.23E-05
9	THBS1	1.167	2.61E-08	9	ACACB	−0.475	2.99E-05
10	COL5A1	1.371	2.84E-08	10	CYP8B1	−0.429	3.47E-05
11	MMP2	1.376	3.89E-08	11	HADH	−0.516	5.24E-05
12	COL1A1	1.799	4.96E-08	12	PIK3R4	−0.346	5.72E-05
13	BCL2	1.169	5.16E-08	13	ARHGAP35	−0.264	7.08E-05
14	PECAM1	0.653	8.90E-08	14	PNPLA3	−0.565	7.34E-05
15	CTBP2	0.672	1.19E-07	15	CAPN5	−0.453	9.65E-05
16	VIM	1.220	1.49E-07	16	CPB2	−0.304	0.0001
17	PLPP4	1.148	1.63E-07	17	PDE3B	−0.391	0.0001
18	MAP3K1	0.579	2.03E-07	18	ADH4	−0.516	0.0001
19	TIMP2	0.886	2.16E-07	19	LPA	−0.941	0.0002
20	DOCK2	0.732	2.24E-07	20	HMGCS2	−0.498	0.0002
21	LGALS3	1.234	2.83E-07	21	SERPINF1	−0.313	0.0002
22	OGT	0.455	2.94E-07	22	ALDH9A1	−0.284	0.0002
23	CCL19	2.422	3.93E-07	23	AP1S1	−0.231	0.0003
24	CXCR4	1.079	4.97E-07	24	CEBPA	−0.414	0.0003
25	TGFB1I1	1.023	5.37E-07	25	ACSM3	−0.483	0.0004
26	CCN2	1.016	6.45E-07	26	ARG1	−0.368	0.0004
27	COL6A3	0.997	7.57E-07	27	PPARG	−0.256	0.0004
28	CD44	0.912	9.71E-07	28	ALDH3A2	−0.273	0.0004
29	TGFB1	0.815	1.01E-06	29	MMUT	−0.305	0.0004
30	ITGA9	0.581	1.02E-06	30	HSD17B4	−0.303	0.0005

The data is ranked after the p-value. The complete list of significantly downregulated genes is shown in Supplementary Table 5. log_2_FC, log_2_-fold change.

### Immunohistochemical validation of S100A4 and LGALS3 expression

**Fig. 2:**
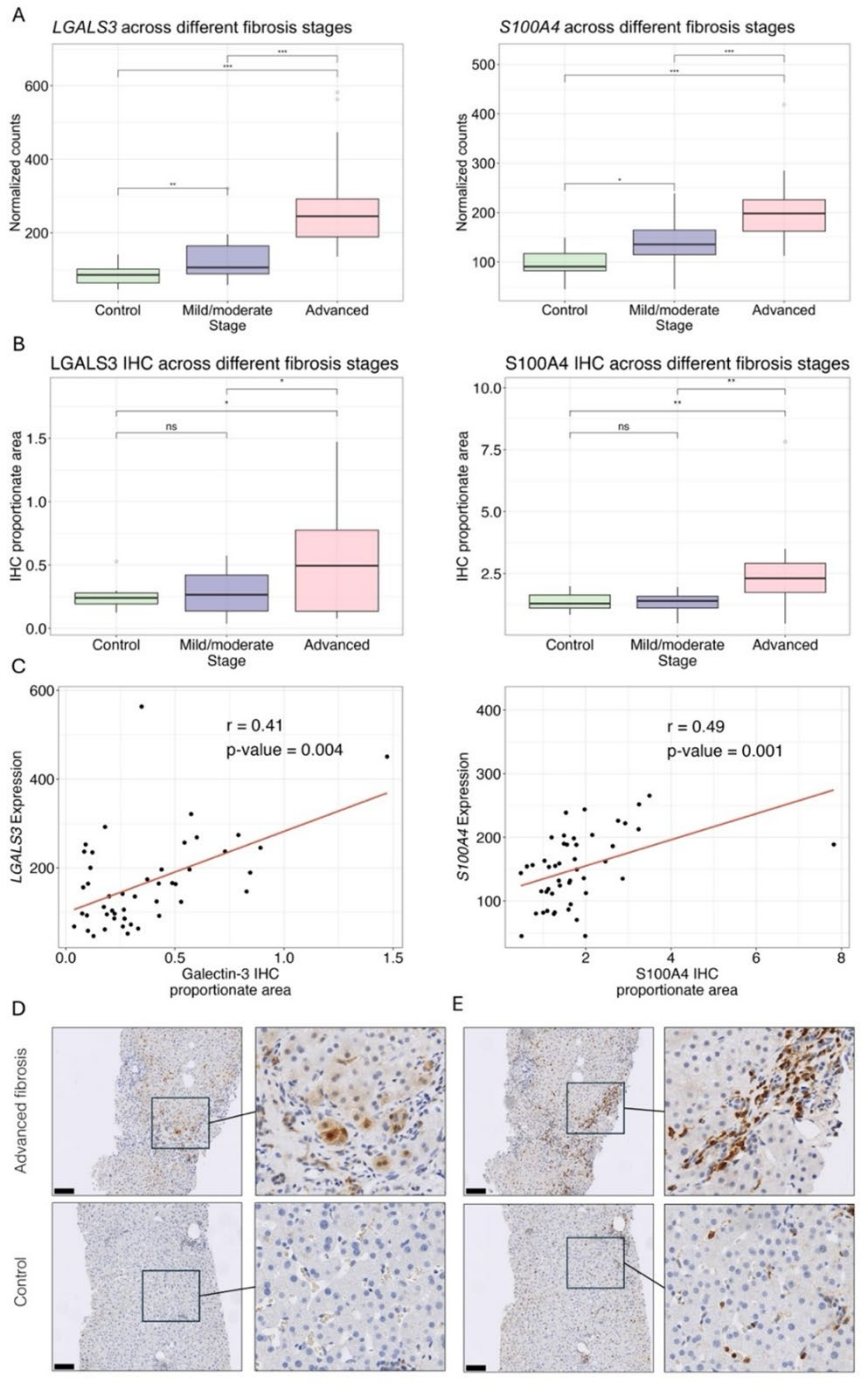
Expression of LGALS3 mRNA (counts) and S100A4 mRNA (counts) and their corresponding proteins assessed by immunohistochemistry and digital image analysis (DIA). (A) Normalized mRNA counts of S100A4 and LGALS3 for mild/moderate fibrosis, advanced fibrosis, and controls. (B) IHC expression of S100A4 and galectin-3 for mild/moderate fibrosis, advanced fibrosis, and controls. (C) Correlation scatter plot of LGALS3 mRNA counts with galectin-3 protein expression, and of S100A4 mRNA counts with S100A4 protein expression. (D) Examples of IHC expression of galectin-3 in advanced fibrosis and controls (scale bars: 100 μm). (E) Examples of IHC expression of S100A4 in advanced fibrosis and controls (scale bars: 100 μm)

We performed IHC for S100A4 and galectin-3 to investigate concordance between gene and protein expression across different disease severity groups. There were significant positive correlations between gene expression and IHC quantification (Fig. 2), suggesting that higher mRNA levels correspond to higher expression of these proteins. IHC images (Fig. 2) illustrate this correlation, with stronger IHC expression observed in advanced fibrosis compared to controls, aligning with the gene expression results.

### Gene set enrichment analysis (GSEA)

**Fig. 3:**
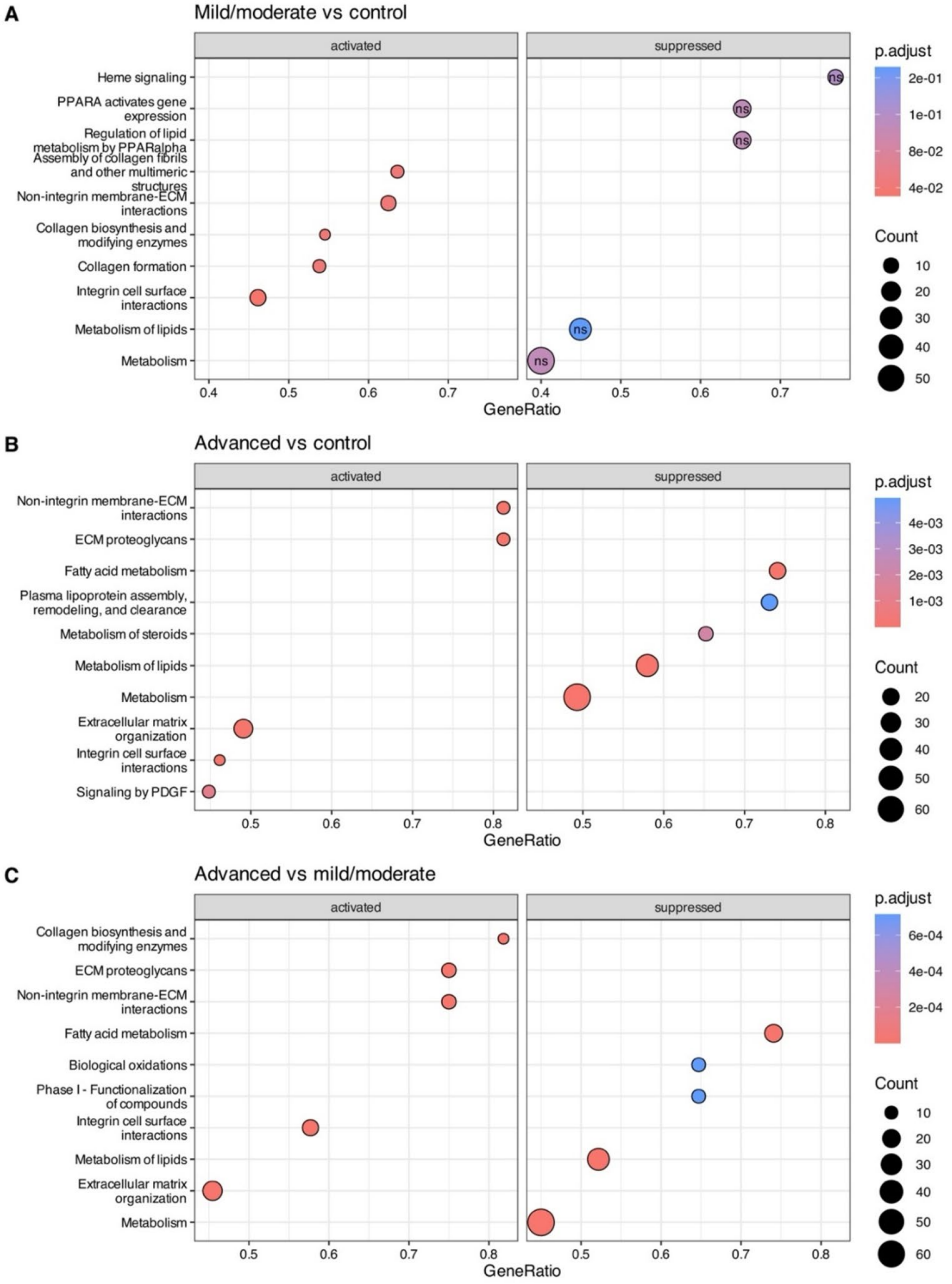
Reactome pathway enrichment analysis with top enriched pathways (sorted by lowest FDR, significance cut-off at < 0.10) showing positive or negative enrichment. (a) Mild/moderate fibrosis vs. control. (b) Advanced fibrosis vs. control. (c) Advanced fibrosis vs. mild/moderate fibrosis. Each dot represents a significantly enriched Reactome pathway, with the x-axis representing the gene ratio (i.e., the proportion of genes in the pathway among the input genes). Dot size denotes the number of genes (count) associated with each pathway. Dot color reflects the adjusted p-value (p.adjust), with red indicating higher significance and blue indicating lower significance. Ns, not significant

The significant DEGs were further analysed to investigate their functional enrichment using *Reactome* gene sets (C2). In mild/moderate fibrosis, top enriched pathways included assembly of collagen fibrils and other multimeric structures (e.g. COL3A1, COL14A1, CTSL), collagen formation (e.g. COL1A1, COL3A1, COL5A1, COL6A3), integrin cell surface interactions (ITGB1, FN1, SPP1), cell cycle, and ECM proteoglycans (Fig. 3, Supplementary Table S6a-c). Additionally, non-integrin membrane-ECM interactions were enriched, indicating early ECM remodeling.

In advanced fibrosis, a pronounced metabolic dysfunction was observed with suppression of pathways involved in lipid metabolism (e.g. ELOVL6, FASN, PPARGC1A) and fatty acid metabolism (e.g. ALDH3A2, ELOVL6, PRKAG2). In contrast, the enrichment of pathways related to fibrogenesis was even more pronounced than in mild/moderate fibrosis. The same pathways that had a tendency toward upregulation in mild/moderate fibrosis were also significantly activated in advanced fibrosis, including assembly of collagen fibrils and other multimeric structures, collagen chain trimerization, collagen formation, biosynthesis and modifying enzymes, and additionally, ECM organization (Fig. 3, Supplementary Table S7a-c).

When comparing advanced fibrosis to mild/moderate fibrosis, the main changes involved suppression of metabolism-related pathways involved in lipid, fatty acid, and steroid metabolism (Fig. 3, Supplementary Table S8a-c), and enrichment of pathways generally related to fibrosis. The total number of significantly enriched or suppressed pathways was 46, similar to advanced fibrosis vs. controls (*n* = 49), while substantially higher than in mild/moderate fibrosis vs. controls (*n* = 8).

## Discussion

In this study, by examining the expression of 760 mRNAs known to be involved in fibrogenesis and inflammation, we delineate a stage-dependent molecular program underlying fibrosis progression in alcohol-related liver disease, moving from early metabolic dysregulation to advanced TGF-β-driven fibrogenic remodeling. By analyzing human liver biopsies across defined fibrosis stages and integrating pathway-focused transcriptomics with protein-level validation, we provide mechanistic insight into how hepatocellular metabolic failure precedes and facilitates fibrotic pathway enrichment.

We observed that certain genes related to glucose and lipid metabolism (e.g., MLXIPL, FURIN, AKT1, INSR) were significantly down-regulated already in mild/moderate fibrosis. MLXIPL regulates carbohydrate-responsive lipogenesis and protects against lipotoxic stress, while FURIN controls the maturation of multiple metabolic and growth-related proteins [28, 29]. Their suppression suggests an early loss of hepatocellular metabolic adaptability, which may lower the threshold for ECM accumulation. However, when evaluating pathways using GSEA, no significant downregulation of broader metabolic pathways was evident in mild/moderate fibrosis, where pathways involved in ECM formation dominated, indicating that while some metabolic genes are affected early, a coordinated suppression of metabolic pathways more broadly emerges only in advanced fibrosis. In advanced fibrosis, we found broader transcriptional changes, including upregulation of genes related to TGF-β1 signaling, ECM remodeling, and inflammation, alongside suppression of metabolic regulators. When comparing advanced to mild/moderate fibrosis, progression was marked by continued downregulation of lipid and fatty acid metabolism pathways, together with enhanced enrichment of fibrogenic programs.

The observed downregulation of metabolic pathways in advanced fibrosis likely reflects the summed effects of alcohol-related hepatocyte injury and the architectural disruption caused by fibrotic tissue, while in mild/moderate fibrosis, fibrogenic activation occurs already while hepatocyte metabolic function remains relatively intact. Next, genes involved in ECM remodeling and pro-fibrotic signaling (e.g., TGFB1, COL1A1, TIMP1) showed progressive up-regulation in more advanced stages, suggesting a shift in balance between matrix deposition and degradation known to characterize hepatic fibrosis [5, 30]. TGFB1 is a master regulator of extracellular matrix production, activating HSCs to produce collagen and other matrix components, and it has been repeatedly shown to be upregulated in human liver fibrosis [5, 31, 32]. In our results, TGFB1 was a key component within many of the significantly enriched pathways and also significantly upregulated in advanced fibrosis compared to mild/moderate fibrosis, thus showing its persistent central role in fibrogenesis during ALD progression. Moreover, our findings suggest that the induction of collagen type I progressively increases in the disease course of ALD, reflecting a shift from metabolic dysfunction and mild/moderate inflammation towards more pronounced fibrotic remodeling. Indeed, several studies have documented a stage-dependent increase of collagen types I, III, and VI in human ALD liver tissue, showing its important role in transition from early to advanced fibrosis stages [33, 34].

Furthermore, in advanced fibrosis, we identified a pronounced metabolic dysfunction with suppressed key pathways involving, e.g., lipid metabolism and fatty acid metabolism. This is consistent with proteomic analyses of human cirrhotic livers, where Niu et al. reported that fatty acid and other metabolic pathways are markedly downregulated, reflecting impaired hepatocellular metabolic capacity [35]. Our findings also align with recent lipidomic studies in ALD showing selective depletion of sphingolipids and phosphocholines with increasing fibrosis stage [36]. Notably, reduced sphingomyelins were associated with liver-related events during follow-up, emphasizing that lipid pathway dysfunction is not merely a molecular signature but also carries prognostic significance. Similarly, low plasma sphingolipids have been linked to poor survival in alcohol-related cirrhosis [37]. Together, these observations support the view that impaired lipid metabolism represents a key feature of advanced ALD and may contribute to both fibrogenesis and clinical outcome.

Furthermore, our data highlights genes involved in inflammation that are upregulated with disease severity, and while IL-8 (CXCL8) did not meet predefined filtering criteria, exploratory analysis suggested increased expression in advanced fibrosis, consistent with its role in inflammatory activation in late-stage disease [27]. On the other side, LGALS3 (galectin-3) and S100A4 were validated at the protein level by IHC. Galectin-3 is a β-galactoside-binding lectin expressed in a wide variety of human cells, and it has been implicated in macrophage activation, fibroblast survival, and amplification of TGF-β–dependent signaling [38, 39]. It has been confirmed to play a role in fibrogenesis in human liver [40] and has also been suggested as a potential biomarker for the severity of fibrosis, while in ALD, it has been shown to be overexpressed in advanced cirrhosis, where it correlated with disease severity and worse outcomes, and discriminated severe alcoholic hepatitis with high accuracy [41, 42]. Cervantes-Álvarez et al. [43] also reported stronger galectin-3 IHC staining in cirrhotic livers, extending beyond Kupffer and bile duct cells to hepatocytes and cholangiocytes, with significantly higher galectin-3–positive areas in advanced disease. This aligns with our findings, where galectin-3 protein expression became more pronounced in advanced fibrosis. Moreover, studies have shown that pharmacological inhibition of galectin-3 attenuates fibrosis in animal models, suggesting that targeting the galectin-3–TGF-β receptor interaction may represent a promising therapeutic strategy to reduce fibrogenesis in ALD [44]. Direct tissue-level assessment by IHC supports the role of galectin-3 as a marker of the pro-fibrogenic inflammatory environment in ALD and may still provide valuable information for clinicians [43].

Similarly, S100A4 (also known as fibroblast-specific protein 1) was among the top up-regulated genes in advanced fibrosis. S100A4 is a calcium-binding protein associated with various cells, including fibrogenic fibroblasts, but in the liver, studies have demonstrated that it is secreted by a subpopulation of macrophages during the progression of liver fibrosis, where it activates HSCs through c‑Myb signaling, leading to increased α‑SMA and ECM production [45, 46]. Our analysis confirms increased S100A4 mRNA levels with advancing fibrosis in ALD. We validated this finding further on the protein level by DIA of IHC, showing accumulation of S100A4 in fibrotic biopsies. Interestingly, Yuan et al. [47] highlighted a dual role in the early alcohol-associated liver injury for S100A4 in mouse models, including suppression of steatosis through the STAT3 pathway and promotion of inflammation. These observations could suggest a stage-dependent role of S100A4, possibly showing a protective effect early on, but perpetuating fibrosis once it sets in.

Our study has several strengths, but also limitations. Using human liver biopsies across a spectrum of ALD severity, we could directly link transcriptomic changes to histologically confirmed fibrosis progression, rather than relying on cell or animal models. Approaches dividing patients into mild/moderate (F1–F2) and advanced (F3–F4) fibrosis groups have been reported in RNA-seq studies using FFPE liver tissue in patients with Fontan-associated liver disease and NASH [48, 49], but to our knowledge, no similar transcriptomics data have been published for ALD cohorts. In MASLD, FFPE samples have been used for both targeted multiplexed mRNA panels and spatial transcriptomics, revealing stage-dependent enrichment of immune and fibrogenic programs and demonstrating that reliable transcriptomic data can be obtained from archived clinical samples [12, 50]. However, to our knowledge, systematic transcriptomic analyses of ALD across different stages of fibrosis severity had not yet been performed.

Our design captured expression shifts across evolving fibrosis groups, offering new insights into ALD progression. Additionally, IHC in the same samples validated key gene expression changes at the protein level. Although the gene panel that we used has the advantage of high reproducibility and sensitivity in FFPE samples [19], one important limitation of our approach is the targeted gene panel, which leaves some aspects of ALD biology underexplored. Additionally, background-based filtering, while reducing false positives, may exclude biologically relevant but low-abundance transcripts, particularly cytokines (e.g., IL-8 (CXCL8)). This may partly explain the relatively low number of DEGs found in mild/moderate fibrosis compared to control biopsies and likewise, the limited power of GSEA. However, this could also be a result of transcriptomic differences being modest between mild/moderate fibrosis and controls. Additionally, bulk-tissue profiling lacks cell-specific resolution, and while our IHC validation provided some context, we could not fully determine the exact cellular sources of transcriptomic changes.

In conclusion, our study provides a multiplexed, targeted, and robust view of the progressive gene expression changes in ALD, spanning from normal to mild/moderate to advanced fibrosis. Our data confirm that metabolic dysfunction emerges early at the gene level, but global metabolic pathway suppression only becomes evident in advanced fibrosis. Concurrently, a coordinated upregulation of pro-fibrogenic mediators—particularly TGF-β1 signaling and collagen deposition—characterizes the fibrotic progression of ALD. Future studies leveraging single-cell approaches might further refine these insights and clarify the precise cellular contributors of these transcriptional changes.

## Electronic Supplementary Material

Below is the link to the electronic supplementary material.

Supplementary Material 1

## Data Availability

The datasets generated during this study are available from the corresponding author on reasonable request.

## Abbreviations

ALD: alcohol-related liver disease
DEG: differentially expressed gene
ECM: extracellular matrix
FFPE: formalin-fixed, paraffin-embedded
GSEA: gene set enrichment analysis
IHC: immunohistochemistry
LGALS3: Galectin-3
MASLD: metabolic dysfunction–associated steatotic liver disease
HE: hematoxylin-eosin
S100A4: S100 calcium-binding protein A4

## Declarations

### Generative AI and AI-assisted technologies in the manuscript preparation process

During the preparation of this work, the authors used AI software (Microsoft Copilot, Microsoft) to proofread and improve the text’s readability. After using this software, the authors reviewed and edited the content as needed and take full responsibility for the content of the published article.
